# A narrative review of current and emerging MRI safety issues: What every MRI technologist (radiographer) needs to know

**DOI:** 10.1002/jmrs.546

**Published:** 2021-09-09

**Authors:** Lisa Mittendorff, Adrienne Young, Jenny Sim

**Affiliations:** ^1^ Department of Anatomy and Medical Imaging, School of Medical Sciences The University of Auckland Auckland New Zealand; ^2^ Mercy Radiology, Silverdale Auckland New Zealand; ^3^ Department of Medical Imaging and Radiation Sciences, School of Primary and Allied Health Care Monash University Clayton VIC Australia

**Keywords:** Magnetic Resonance Imaging < Discipline, Adverse Events < General, Contraindications < General, Research ‐ review < General, Radiographer < Medical Imaging, Medical Imaging < General, Patient care < General

## Abstract

Magnetic resonance imaging (MRI) has been traditionally regarded as a safe imaging modality due to the absence of ionising radiation. However, MRI is a source of potential hazards with a variety of risks including, but not limited to, those associated with the various electromagnetic fields used for imaging. All MRI technologists (radiographers) require sound knowledge of the physical principles of the MRI scanner and must understand the associated safety risks and how to avoid adverse events from occurring. MRI technologists now assume more responsibility in clinical decision‐making, and their knowledge base has consequently had to expand significantly. In addition, rapid advancements in MRI technology and other correlated areas such as medical implant technology, and the associated increase in MRI safety issues, place increasing demands on the MRI technologist to constantly keep abreast of current and future developments. This article reviews current and emerging MRI safety issues relevant to the three MRI electromagnetic fields and highlights the key information that all MRI technologists should be fully cognisant of to ensure competent and safe practice within the MRI environment.

## Graphical abstract

Rapid advancements in MRI technology and other correlated areas such as medical implant technology, and the associated increase in MRI safety issues, places increasing demands on the MRI technologist to maintain up‐to‐date knowledge and to constantly keep abreast of future developments. This article reviews current and evolving MRI safety issues and summarises the key information that all MRI technologists should be fully cognisant of to ensure competent practice and the safety of patients, staff and other personnel within the MRI environment.

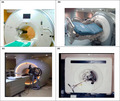

## Introduction

With its ability to provide exceptional soft tissue contrast, combined with non‐ionising radiation exposure, magnetic resonance imaging (MRI) has become widely used in medical imaging globally.^1^ However, the three electromagnetic fields used in MR imaging (the static magnetic field, the radiofrequency field and the time‐varying gradient magnetic fields)^2^, ^3^ each create their own safety risks that MRI technologists must have appropriate understanding of to ensure adverse events are avoided.^4^, ^5^ Projectile forces, biomedical implant and device risk, heat deposition and acoustic noise all have the potential to cause significant harm or even death.^2^

It is evident that as MRI technology evolves and clinical practice becomes more complex, so too do the associated safety issues.^6^ The role of the MRI technologist is also evolving, and comprehensive and up‐to‐date knowledge of MRI safety issues is essential to ensure safe care. This article provides an overview of MRI safety issues that MRI technologists must understand and be capable of managing to ensure safe clinical practice.

## Static magnetic field (B_0_)

MRI system software and hardware have evolved considerably over the last three decades. Until recently, the main magnetic field strength of most clinical scanners was 1.5 Tesla (T) although the installation of high‐field 3T systems has now become commonplace in the clinical setting. These stronger magnets have the potential to deliver improved efficiency and increased image quality but introduce additional safety risks within the MRI scanner room.^1^ These risks include the potential for a higher number of projectile incidents.^7^, ^8^

The U.S. Food and Drug Administration (FDA) has deemed static magnetic field strengths up to 8T to be safe for use in humans aged more than 1 month, opening the way for ultra‐high‐field MRI systems (7T and above) to become increasingly utilised in clinical practice.^1^ Although these systems are currently generally confined to clinical research use, in October 2017, the FDA approved the use of the first 7T scanner for clinical application at the Mayo Clinic in North America.^9^ Worldwide, there has been a rapid rise in 7T systems now being used, with over 60‐80 scanners in both clinical and research settings.^7^, ^10^

### Safety issues associated with B_0_

Clinical MRI scanners commonly utilise a superconducting magnet, so consideration must be given to the fact that the static magnetic field is always present, as are the associated safety hazards.^11^

#### Ferromagnetic objects, implants and devices

**Figure 1:**
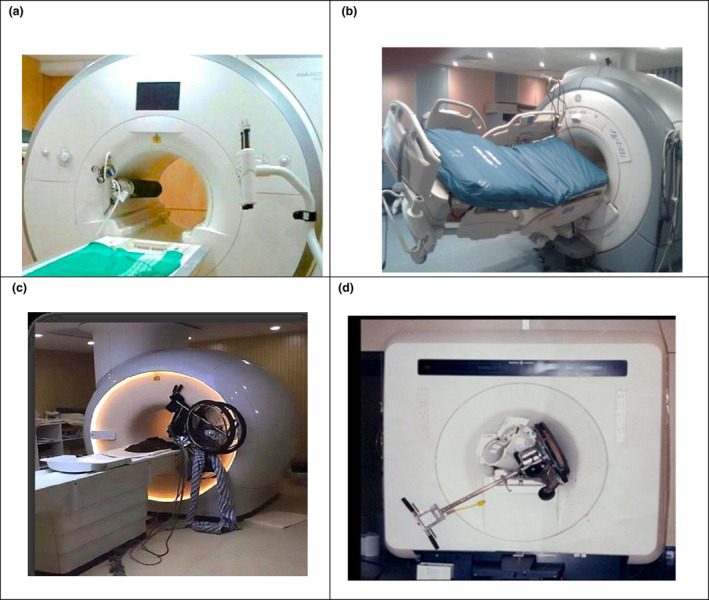
Examples of projectile accidents in the MRI environment: (A) oxygen cylinder, (B) stretcher bed, (C) wheelchair, (D) floor buffer. (Images courtesy of, and reprinted with permission from, Frank G. Shellock, Ph.D., www.MRIsafety.com).

In 2018, Song et al.^12^ estimated that 10 to 20% of all MRI patients have implanted medical devices. Ferromagnetic objects, including medical implants and devices, are subjected to both translational and rotational forces when introduced to the static magnetic field.^13^ The projectile (or missile) effect is caused by the translational force, whereby these objects are violently drawn into the magnet bore.^14^ This force increases the closer the object gets to the magnet and is defined by the object’s magnetic susceptibility, with ferromagnetic objects having very high, positive magnetic susceptibility.^11^, ^15^ Translational force is associated with the numerous injuries and fatalities reported in incidents involving objects ‘flying’ into the magnet such as oxygen cylinders,^16^ stretcher beds,^17^ wheelchairs^18^ and floor buffers^19^ (Fig. 1A‐D).

**Figure 2:**
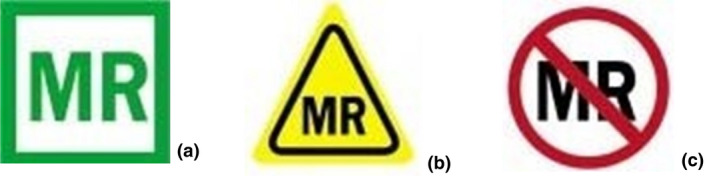
Icons used in the ASTM F2503‐20 standard for MR safety category labelling: (A) MR Safe, items that pose no known risk or hazard in the MRI environment; (B) MR Conditional, implants and devices demonstrate no hazard in the MRI environment, but only when prescribed conditions for safe use are adhered to; (C) MR Unsafe, implants and devices should never be brought into the MRI environment.^21^

Despite these risks, it is important that patients with medical implants and devices are not unnecessarily excluded from MRI. Having an accurate understanding of the composition of these objects and their behaviour in the MRI environment can enable such patients to undergo an MRI examination.^20^ To assist this, the American Society of Testing and Materials (ASTM) International Committee designed a labelling system in 2005 that identifies three MRI safety categories: MR Safe, MR Conditional and MR Unsafe, each with an associated icon (Fig. 2).^20^, ^21^

Conditions defined for MR Conditional items usually include the strength of the static magnetic field (B_0_) and spatial gradient magnetic field (SGMF) limits.^3^ B_0_ extends three‐dimensionally beyond the actual magnet bore and will vary in strength dependent on distance from the scanner. This change in intensity relative to distance is known as the spatial gradient magnetic field (dB/dx) and is measured in T/m or Gauss/cm, where 1T/m equals 100G/cm.^11^, ^22^ Translational force is proportional to dB/dx and is therefore strongest at the point where the SGMF is the greatest. This point is typically at the entrance to the scanner bore.^22^

**Figure 3:**
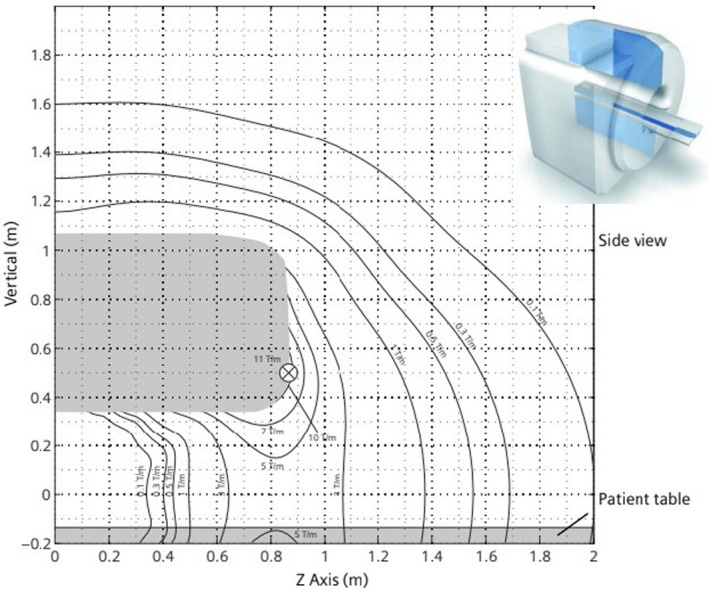
Example of vendor spatial gradient magnetic field map: 3T Skyra scanner. (Courtesy of Siemens Healthineers).

MRI system vendors provide SGMF maps for each scanner, which plot the change of the static magnetic field over distance and demonstrate the point of maximum spatial gradient (Fig. 3). Vendors have their own SGMF maps, and these will differ between scanners, requiring the MRI technologist to be able to interpret different SGMF map formats. MRI technologists must be capable of interpreting these maps to determine implant safety with respect to any defined spatial gradient limitations prior to imaging patients with medical devices.^20^, ^22^

Ferromagnetic objects also experience a rotational force (torque), when brought into the scanner room, and this force is greatest in the centre of the bore. Objects are forced to rotate to align with the direction of the main magnetic field.^15^ The combination of translational and rotational forces may result in harm to a patient or death as it can cause implant dislodgement, mechanical failure in active implants, or movement of metallic devices or foreign bodies. Related cases reported in the literature include aneurysm clip displacements,^23^ cardiac pacemaker failures^24^ and drug infusion pump malfunctions leading to death.^25^ Furthermore, orbital injuries have occurred where an undetected intraorbital foreign body has resulted in blindness.^26^, ^27^

As the potential to injure is high if ferromagnetic objects are inadvertently brought into the MRI scanner room, recent updates by the American College of Radiologists (ACR) Committee on MR Safety recommend the door to the scanner room always remains closed, unless for patient access or system maintenance. Furthermore, it is suggested that if the door is open, a plastic chain be utilised as a ‘caution’ barrier to prevent unauthorised access into the scanner room.^6^

Obtaining a thorough patient medical history is also necessary to identify any previous surgery and/or injuries that need to be assessed prior to an MRI examination.^28^ Should any implants or devices be noted, the MRI technologist must be aware of international guidelines and local workplace policies to ensure that best practice is adhered to and safety is not compromised. For example, one radiographic view of the orbits has been deemed to be sufficient to provide adequate information to either rule out, or provide an anatomical position of, any foreign body within the orbit.^6^

**Take home points:** As technology evolves, patients are presenting with an ever‐increasing variety of implantable devices and the MRI technologist must be capable of obtaining the most relevant information regarding the safety of the device. Interpretation of any MR conditions (including B_0_ and dB/dx limitations) is also essential, as is the ability to apply these conditions to a specific scanner in the workplace by understanding the vendor SGMF maps.

#### Bioeffects

Many biological processes have been investigated in relation to B_0_, but no conclusive proof of harmful biological effects has been found to be caused by the static magnetic field up to 7T.^2^, ^7^, ^11^ This includes a lack of evidence to suggest any harm associated with MRI during pregnancy.^29^

Nevertheless, reports indicate findings of acute sensory effects, including a metallic taste, magnetophosphenes, nausea and vertigo, associated with moving through the static magnetic field.^6^, ^25^, ^26^ Suggested to be related to the induced voltages created by this movement (due to Faraday’s law of induction),^30^ these effects are of particular concern as 7T systems are introduced into the clinical setting, because preliminary research has demonstrated they are more evident at higher field strengths.^8^ The MRI technologist needs to be aware that encouraging the patient to keep their head still while moving through the magnetic field, particularly when utilising a 7T system, can assist with reducing the potential adverse effects such as vertigo and nausea.^7^

## Radiofrequency field (B_1_)

While to date, the static magnetic field is accountable for all MRI fatalities, the radiofrequency (RF) transmit subsystem, used to excite the patient’s tissues and produce the MR signals required for image acquisition, is responsible for the highest number of reported adverse injuries, namely burns.^2^, ^31^As high‐field and ultra‐high‐field MRI scanners are more widely implemented clinically, the associated higher frequencies of the B_1_ field may affect tissue heating and introduce further MRI safety issues such as increased power deposition and related concerns over RF burns, unpredictable specific absorption rate (SAR) control, and implant and device heating.^7^, ^8^ The development of new techniques, such as parallel transmission that utilises multiple RF transmit coils, also raises new safety concerns.^32^

### Safety issues associated with B_1_

#### RF burns

Burns have been consistently identified as the most common type of MRI adverse event^4^ and, in 2019, it was reported that 59% of the FDA’s MAUDE MRI adverse event database was related to thermal injury.^33^ Burns caused through MRI scanning are predominantly caused by electrically conductive materials introduced into the scanner, direct contact with RF coils, proximity burns from contact with the scanner bore itself or an electrical loop formed by the patient’s body. ^23^, ^33^

**Figure 4:**
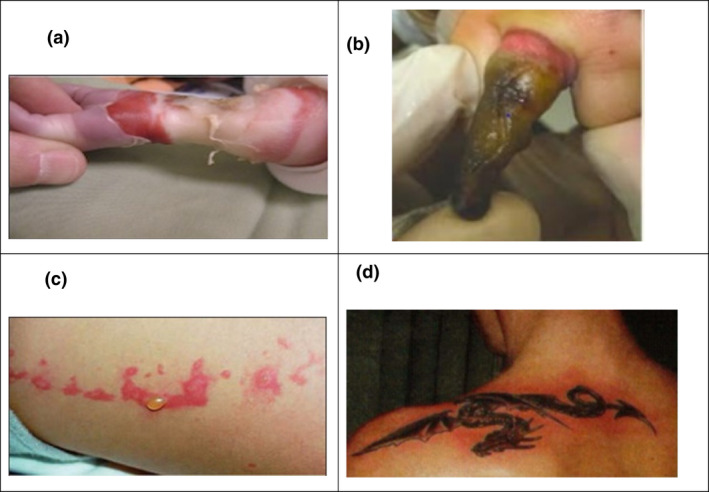
Examples of RF induced burns: (A) fourth‐degree burn immediately after MRI examination on a 5‐week‐old baby, caused by an MR Unsafe pulse monitor. This resulted in amputation of the forearm^58^ (B) burn resulting from an MR Unsafe pulse oximeter on the finger, post‐escharotomy (C) second‐degree burn after MRI examination from invisible metallic microfibres in clothing (D) first‐degree burn around a tattoo after MRI. (Images courtesy of, and reprinted with permission from, Frank G. Shellock, Ph.D., www.MRIsafety.com).

If equipment comprised of conductive materials such as physiological monitors and electronically controlled devices remain in the magnet bore with the patient, it is possible for localised heating and burns to occur where they are in direct contact with the skin.^2^ Two recent case studies reported by Abdel‐Rehim, Bagirathan et al.^34^ demonstrated that placement along the patients’ abdomens of insulated electrocardiograph (ECG) monitor cables (described by the authors as ‘MRI compatible’) created full‐thickness burn lines. Both patients were reported as having a high body mass index and had been subjected to a lengthy MRI examination, whereby the possibility for the lack of thermal regulatory ability and the formation of conductive loops of the cable could have been responsible for the burn. Figure 4A and B further demonstrates the consequences of using MR Unsafe equipment during the MRI examination.

Skin‐to‐skin contact can also lead to a high current concentration, which results in focused and localised heating, ultimately leading to RF burns.^2^ The skin itself is conductive, and during RF deposition, a current is induced at the area of highest resistance (such as where a closed loop is formed by fingers and thighs touching), producing heat substantial enough to cause tissue damage.^35^ Reports confirm that improper patient positioning can result in burns, as in the case of a young man who received second‐ and third‐degree burns to the insides of his thumbs and index fingers where they had been in contact with each other.^35^ Likewise, in 2017, a patient developed blisters on the inside of both thighs during an MRI examination, resulting in second‐degree burns where the thighs were touching.^36^

Novel MRI burn hazards are also being created with technological advances in other industries, as in the athletic clothing manufactured with invisible silver‐embedded microfibres. This conductive material can produce electromagnetic eddy currents, and there have been multiple reports of second‐degree burns to patients when scanned while wearing such clothing^31^, ^37^ (Fig. 4C). This highlights the importance of changing all patients into facility‐provided gowns.

More recently, with COVID‐19 necessitating the mandatory wearing of face masks during MRI examinations, MRI technologists need to understand the importance of checking masks for any metallic component. In 2020, an MRI face mask burn was reported,^38^ reinforcing the need to remain informed of emerging issues and vigilant during the patient screening and preparation processes.

Similarly, several transdermal patches such as those used to administer pain relief and nicotine patches may have a metallic backing.^39^ This foil backing can induce an electrical current at the skin surface and act as a conductor when the RF is generated, increasing the risk of heating and burns.^39^ Such a case was reported in 2004 when a patient with a nicotine patch suffered second‐degree burns during an MRI.^40^ The United Kingdom (UK) Medicines and Healthcare Products Regulatory Agency (MHRA) recommend removal of medicinal patches that may contain metal, as long as patient treatment would not be compromised, to minimise risks associated with patch heating such as burn injuries or even a life‐threatening overdose of medication such as fentanyl.^41^

Tattoos and permanent cosmetic products that contain metallic components can also pose a risk. In 1996, discussion regarding the risk of permanent cosmetics began after a patient with tattooed eyeliner underwent an MRI examination. The reaction of the iron oxide pigments to the RF magnetic field was thought to have caused painless oedema for 48 hours after the MRI.^41^ Ross et al.^42^ reported a case study in 2011 where a potential electric current was produced in metallic tattoo pigments on bilateral thigh tattoos, producing a cutaneous burn at each tattoo site. Figure 4D demonstrates a first‐degree burn on a shoulder tattoo after MRI.^19^

Shellock et al.^13^ investigated burning or heating associated with tattoos used for permanent cosmetics, but determined that the actual risk of any incident occurring was fairly remote and should not negate an MRI if needed. They suggested that a cold compress may be applied to minimise any risk to the patient and that the risk is far greater to the patient in the long term if a clinically important MRI procedure was cancelled. The FDA agrees that adverse tattoo incidents appear to be rare and have no long‐lasting effects.^43^

**Take home points:** MRI technologists must understand how burns are caused and adhere to best practice by implementing preventative measures such as: changing the patient out of street clothes, avoiding skin‐to‐skin and skin‐to‐bore contact with the use of pads, checking the patient’s arms or legs are not crossed, ensuring no leads or monitors come into contact with the patient’s skin and using a heat sink over tattoos situated within the RF coil (Table 1).

**Table 1:** Examples of how to reduce the risk of burns.

	Preventative measure
Physiologic monitors, leads and cables	Leads or cables should not directly contact the patient; place pads at least 1cm in thickness (not a sheet) between the patient and lead/cable. All equipment within the scanner room must be MR Conditional.^41^
Skin‐to‐skin contact	Place pads at least 1cm in thickness (not a sheet) between places where there may be skin‐to‐skin contact within the RF field, for example between the thighs or where the thumb may touch the side of the body. To avoid a conductive loop, the legs and arms should not be crossed.^41^ 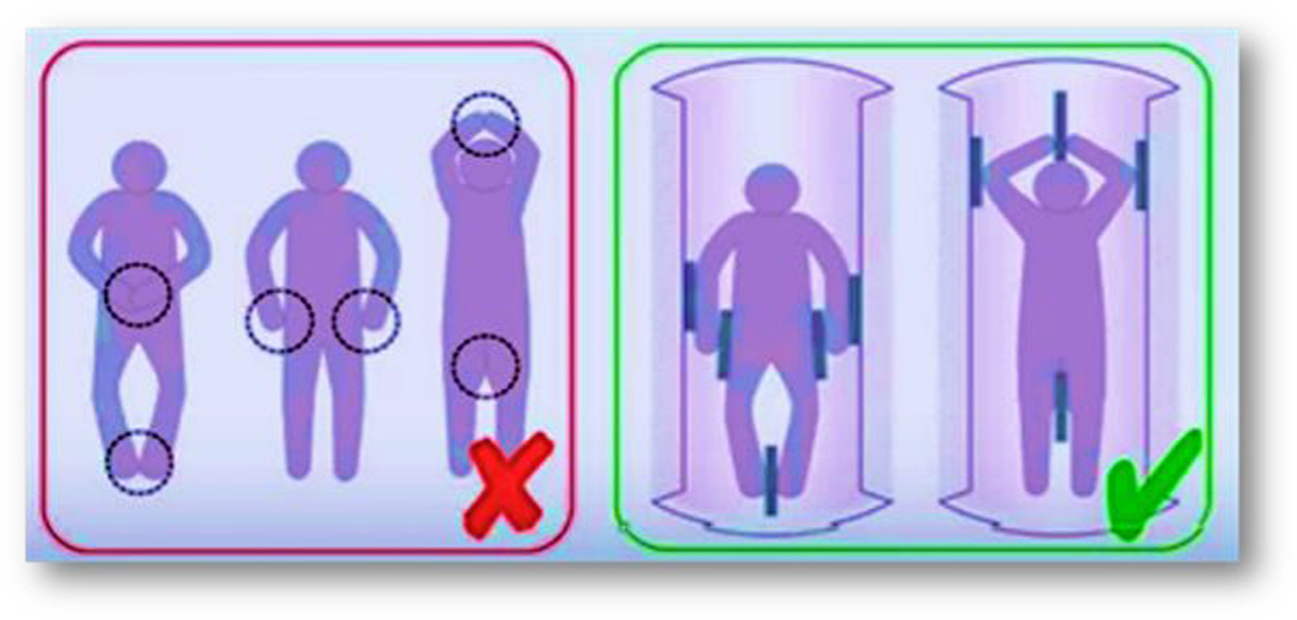 **(Image courtesy of Siemens Healthineers)**
Skin‐to‐magnet bore contact or skin‐to‐transmit RF coil contact	Ensure pads at least 1cm in thickness (not a sheet) are placed between the patient and the side of the bore, or the patient and RF transmit coil if contact is possible.^41^
Metallic microfibre clothing	Change all patients out of street clothes into MRI safe gown or scrubs.^6^
Face masks	Remove metal nose piece from mask where necessary.^38^
Tattoos	Place a cold compress over the area of interest to provide a heat sink.^6^

#### Implant Heating

It has been demonstrated that objects made of conductive material that have an elongated shape, for example lead wires, catheters and electrodes, and are of a specific length that causes resonance with the RF field have a high risk of heating.^2^, ^13^ Known as the antenna effect, the potential current induced in such objects when exposed to the RF field can cause considerable heating, particularly in biological tissues surrounding their tips.^30^

A recent study by Song et al.^12^ specifically investigating the relationship between heating and passive medical implants confirms that consideration needs to be made with regard to implant length and the frequency of the RF field (which is related to the static magnetic field strength) when assessing the safety of such implants.^12^ Table 2 displays lead length risk for specific magnet strengths. Taking this into account, in some cases, it may be safer to scan a patient at 3T than it is to scan at 1.5T, depending on the length of the implant.

**Table 2:** Risk of heating of specific lead lengths at different magnetic field strengths.^8^, ^30^

Magnet strength	RF	Lead length risk
1.5T	64MHz	25‐30cm
3T	128MHz	10‐15cm
7T	298MHz	5‐7cm

Specific examples of thermal tissue damage in MRI caused by implanted medical leads and electrodes have been reported in several case studies. In 2003, Spiegel et al.^44^ examined the incident of a 73‐year‐old patient with implanted bilateral deep brain stimulator (DBS) electrodes, who immediately after MRI of the brain, suffered dystonic and partially ballistic left leg movements. A more recent case reported in 2012 described thermal brain injury occurring in a patient with an MR Conditional intracranial pressure (ICP) monitoring device when the tip of the monitor’s fibre‐optic transducer melted, subsequently causing a deep white matter injury.^45^ These examples of significant injury emphasise the importance of understanding the causes of implant heating and have brought about an increased awareness of the importance of adhering to manufacturer guidelines and performing MRI examinations under well‐defined conditions.^46^

#### Bioeffects

RF deposition during an MRI examination produces thermogenic effects within the patient, which could possibly lead to physiological changes.^11^ It is important that the MRI technologist understands the implications of regulations designated by the International Electrotechnical Commission (IEC) for reducing SAR effects and the limits per examination on predicted increase in body temperature (Table 3).^3^, ^47^

**Table 3:** International Electrotechnical Commission’s SAR regulations^3^, ^47^ and examples of how to reduce SAR.^11^, ^49^

Operating Mode	Whole‐Body SAR limit (W/kg)	Head SAR limit (W/kg)	Risk of physiological stress to subjects	Requirements
Normal	2	3.2	Unlikely to cause physiological stress	None – used for routine scanning
First‐level controlled	4	3.2	May cause physiological stress	Needs to be controlled by medical supervision
Second‐level controlled			Significant risk	Explicit ethical approval is required according to local requirements

SAR reduction method	Trade‐offs
Change patient into light gown/scrubs	Some patients may object
Control room temperature to keep cool	Patient may feel the cold more
Reduce the slice number per acquisition	Longer scanning time
Reduce the flip angle	Could alter contrast and signal to noise ratio
Alternate high and low SAR sequences/have breaks between high SAR sequences	Possible longer scanning time
Reduce pulse frequency (use normal or low SAR options)	Longer scanning time
Increase TR	Longer scanning time
Remove saturation bands	Increase in image artefact
Remove Restore pulse	Increase in TR increases scanning time
Reduce echo train length in (turbo factor)	Longer scanning time

Many factors contribute to the SAR level reached during an MRI examination. These include the frequency of the RF field, size of the anatomical region within the imaged volume, type of RF coil used, repetition time (TR) and even the orientation of the patient within the magnet bore.^13^ Schaefer^48^ further proposes that patient temperature and degree of sweating, combined with ambient room humidity and airflow, all contribute to the extent of patient heating during an MRI examination. Increased age^49^ and clinical conditions such as hypertension, cardiovascular disease, obesity, diabetes or the use of various medications may also alter the patient’s response to heat overload and their ability to regulate their body temperature.^13^

Various technical parameters can be manipulated during an MRI examination that will directly alter SAR values (Table 3).^11^, ^49^ This follows that the MRI technologist has a degree of control over the amount of heat deposited.^11^

There has also been some concern regarding the potential effect of heating on the foetus if a pregnant patient is scanned. However, a 2015 study of over 1700 women who had an MRI in their first trimester of pregnancy demonstrated no increased risk of harm to the foetus from heating.^50^ Nevertheless, it is recommended that only Normal Operating Mode should be used during pregnancy, and parallel transmission is not used until further studies have been conducted.^3^

**Take home points:** The technologist should be mindful of the patient’s age, status of their thermoregulatory system and any other underlying health condition that may compromise their ability to disperse or tolerate heat change. MRI technologists must be capable of altering environmental and scanning parameters to minimise patient heating. They must also understand why specific scanner field strengths, RF coils, SAR limitations and lead positions are defined for various MR Conditional implants and devices and the potentially adverse impact of not adhering to these conditions.

## Time‐varying gradient magnetic fields

To localise MRI signals, small linear magnetic field gradients are applied during image acquisition using three gradient coils. These coils are rapidly switched on and off, and when in the presence of the strong static magnetic field, Lorentz forces are generated causing a physical vibration of the gradient coils against their mountings.^51^ This vibration is responsible for the loud noises created when scanning.^52^ These time‐varying gradient fields are also capable of inducing a number of biological effects including peripheral nerve stimulation (PNS) and magnetophosphenes.^13^ With the evolution of ultra‐high‐field MRI systems and the potential use of stronger gradients to overcome issues with associated susceptibility effects,^43^ a corresponding increase in these safety risks is of concern.^8^

### Safety issues associated with the gradient magnetic fields

#### Acoustic noise and hearing damage

During the MRI examination, the patient, and any support personnel who remain in the scanner room, is exposed to the gradient magnetic fields.^52^ Early reports of consequent hearing loss and tinnitus have established the associated acoustic noise as a specific MRI hazard.^30^, ^33^ The IEC requires hearing protection to be used when acoustic threshold exposure limits exceed 99dB.^47^ Most MRI systems will exceed this limit, so hearing protection is mandatory and must be correctly used by all those remaining in the MRI scanner room during an MRI examination.^33^, ^47^ With properly fitting earplugs offering only about 20dB attenuation, a combination of earplugs and headphones has been recommended by vendors in recent years.

In 2013, Mollasadeghi et al.^53^ reported a case where a patient suffered bilateral sensorineural hearing loss for three months after a 25‐minute scan on a 1.5T MRI system, when not wearing appropriate hearing protection. De Wilde et al.^4^ reported a similar case in 2007 on a 0.5T system when a patient, also without hearing protection, suffered headaches and hearing loss following the MRI examination. Concerns about scanning pregnant women because of the potential to cause hearing damage in the foetus have also been raised although, in the previously mentioned study of women scanned in their first trimester of pregnancy, there was no evidence of increased risk of harm to the foetus from acoustic noise.^50^

#### Bioeffects

Although hearing damage is more prevalent, PNS is considered the more commonly known biological effect of the time‐varying gradient magnetic fields.^54^ This issue has become the limiting factor to the size and slew rate of the gradient coils that can be used in MRI.^55^ Currently, there are little data on PNS at 7T and opinion is divided as to whether there is an increased risk at the higher field strength.^8^

PNS is generally reported as a slight tingling sensation, although the severity of PNS pain can range from barely noticeable to extreme and is dependent on the subject.^4^ MRI technologists must recognise that there is a higher chance of PNS occurring when rapid sequences such as echo‐planar imaging (EPI) are used, the y‐gradient is selected for frequency encoding and/or high‐resolution images or oblique slices are acquired.^3^ The possibility of inducing PNS can be reduced by avoiding y frequency‐encoding gradients during EPI examinations.^56^

Shellock et al.^13^ report that pain, or in extreme circumstances, cardiac stimulation, can be caused at high‐gradient magnetic field strength. However, as the threshold for cardiac stimulation is extremely high (i.e. 900% of the mean threshold for PNS), cardiac stimulation associated with a diagnostic MRI examination is extremely unlikely as gradient slew rates are restricted to safe levels by the scanner manufacturers.^57^

**Take home points:** The MRI technologist must know how to correctly fit earplugs and check that the patient does this. They must also ensure that appropriate hearing protection is provided to, and worn by, anyone remaining in the scanner room during the examination. To avoid PNS, the MRI technologist must recognise the risk factors, and take preventative measures if necessary, to minimise any discomfort to the patient.

## Conclusions

The evolution of MRI over the last three decades has seen an increase in MRI safety concerns due to advancements in technology and the higher demand for MRI procedures. Irrespective that experience and knowledge regarding potential MRI safety hazards have been gained over this period, the number of adverse incidents appears to be on the rise. The static magnetic field strength of clinical magnets is now routinely 1.5T and 3T, and more recently, the clinical inclusion of 7T scanners increases possible safety concerns.

Medical implants and devices continue to be developed enabling more patients with these to be scanned in MRI, although alongside this comes an increased requirement for MRI technologists to assume responsibility of interpreting MRI‐related conditions. Furthermore, the increased demand on MRI technologists to scan off‐label implants requires an advanced level of understanding of MRI safety conditions to aid in risk‐versus‐benefit analysis. In general, however, it is ultimately the responsibility of the supervising radiologist/physician to make the decision to scan the patient in an off‐label situation.

MRI technologists in New Zealand and Australia now assume more responsibility in clinical decision‐making, and their knowledge base has had to expand. The unique safety risks associated with the MRI environment require the MRI technologist to have a comprehensive understanding of MRI hardware and its underlying physical principles. Rapid advancements in MRI technology, and the rise in associated MRI safety issues, heighten the urgency for all MRI technologists to maintain up‐to‐date MRI safety knowledge. This can only be achieved through ongoing education and training to ensure safe patient care.
